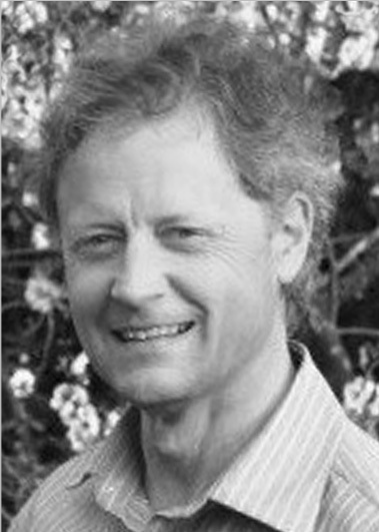# Anion Recognition in Water: Recent Advances from a Supramolecular and Macromolecular Perspective

**DOI:** 10.1002/anie.201506589

**Published:** 2015-11-27

**Authors:** Matthew J. Langton, Christopher J. Serpell, Paul D. Beer

**Affiliations:** ^1^ Chemistry Research LaboratoryDepartment of ChemistryUniversity of OxfordMansfield RoadOxfordOX1 3TAUK; ^2^ School of Physical Sciences, Ingram BuildingUniversity of KentCanterburyKentCT2 7NHUK

**Keywords:** anions, host–guest systems, molecular recognition, supramolecular chemistry, water

## Abstract

The recognition of anions in water remains a key challenge in modern supramolecular chemistry, and is essential if proposed applications in biological, medical, and environmental arenas that typically require aqueous conditions are to be achieved. However, synthetic anion receptors that operate in water have, in general, been the exception rather than the norm to date. Nevertheless, a significant step change towards routinely conducting anion recognition in water has been achieved in the past few years, and this Review highlights these approaches, with particular focus on controlling and using the hydrophobic effect, as well as more exotic interactions such as C−H hydrogen bonding and halogen bonding. We also look beyond the field of small‐molecule recognition into the macromolecular domain, covering recent advances in anion recognition based on biomolecules, polymers, and nanoparticles.

## 1 Introduction

The chemical recognition of anions has progressed in leaps and bounds in the last twenty years or so, during which time a huge array of selective receptors and sensors have been developed. However, the proposed applications are overwhelmingly in the biological, medical, environmental, and industrial arenas and, therefore, typically require aqueous conditions,^1^, ^2^ while the majority of research to date has taken place in organic media (in contrast to the earliest hosts^3^). The design of selective anion receptors that function in aqueous solution is, therefore, a key challenge in supramolecular chemistry: in particular, examples of receptors that are neutral or of low charge and operate in organic–aqueous mixtures are uncommon, and those that function in 100 % water are rarer still. Water itself is a highly competitive polar solvent, able to hydrate the host and guest through hydrogen‐bond donation and acceptance, whilst anions themselves possess intrinsic properties that make them difficult to bind effectively in aqueous solution. Anions are heavily solvated in water, typically more so than analogous cations of the same charge and similar size,^4^ they also display a much greater range of hydrophilicity/hydrophobicity,^5^ and their recognition is further complicated by the multiple protonation equilibria that many exhibit. Furthermore, anions are in general relatively large, thereby leading to weaker electrostatic interactions with a positively charged host. From a synthetic chemistry perspective, the design and synthesis of water‐soluble receptors is also demanding, and requires careful consideration of potential competing self‐assembly processes arising from amphiphilicity. Many such artificial anion receptors suffer from poor aqueous solubility, thus necessitating the use of organic–aqueous solvent mixtures for anion‐binding investigations. Nevertheless, overcoming this range of challenges is essential if applications of synthetic anion receptors in competitive as well as biologically and environmentally relevant aqueous media are to be exploited.

Anion recognition in water has been central to one of the most interesting science stories to hit the mainstream news in recent years. In 2011, it was reported that a strain of bacteria taken from Mono Lake in California (USA) could grow in media rich in arsenate but free of phosphate.^6^ Such a result implied that arsenic could replace phosphorus in the bacterial genome, and would be the first instance of life comprised of alternative elements—thus meaning that the search for life outside Earth should be significantly broadened in terms of possible environments. However, the report was immediately questioned by many in the scientific community on a number of different fronts, and it was discovered that the bacteria possessed such highly specific phosphate‐binding proteins that they could survive on the merest of trace levels of phosphate.^7^ The microorganisms have shown that such precise discrimination is possible in water—now the scientific community is aiming to achieve this level of selectivity with tunable synthetic recognition systems.

Stefan Kubik, a pioneer in the use of neutral host molecules for anion binding in aqueous solutions, has reviewed the state of the art in the field of anion recognition in water using supramolecular host systems, the most recent of which was published in 2010.^8^, ^9^ However, in the past five years numerous exciting and significant advances in this rapidly expanding field have emerged, in particular exotic interactions such as C−H hydrogen bonding and halogen bonding have been exploited in anion host systems capable of operating in water. The hydrophobic effect in particular has attracted much interest in the context of anion recognition in water by electroneutral hosts and, yet, of all of the factors driving host–guest complexation, it is arguably the least well understood. Recent studies have highlighted and investigated the nuanced relationship between the hydrophobic effect, the Hofmeister series, ion–solute interactions, and the thermodynamic contributions that dominate these phenomena, which has been aided by the recent design and synthesis of hydrophobic host systems capable of strongly binding anions in water.

This Review commences with the recent advances in the field of supramolecular anion coordination chemistry in water, from 2010 to the present day. We have chosen to only include examples of synthetic receptors which operate in solvent mixtures containing 50 % or more water, with particular emphasis laid on those capable of functioning in 100 % aqueous solutions. We will also look beyond the field of small‐molecule recognition into the macromolecular domain, covering recent advances in anion recognition based on biomolecules, polymers, and nanoparticles in ≥50 % water. Since this wider perspective has not yet been reviewed, the time constraint is somewhat more relaxed.

## 2 Small‐Molecule Supramolecular Anion Receptors

### 2.1 Charged Hydrogen‐Bonding Anion Receptors

Anion recognition in water by small‐molecular supramolecular receptors has been predominantly the domain of positively charged hosts. Examples include the protonated macrobicyclic anion receptors, described by Park and Simmons in seminal studies,^3^ the quaternary ammonium cages from Schmidtchen,^10^ polyammonium macrocycles, cryptands,^11^ and macrocyclic guanidinium‐based receptors from Lehn and co‐workers,^12^ as well as many more recent examples.

**Figure 1:**
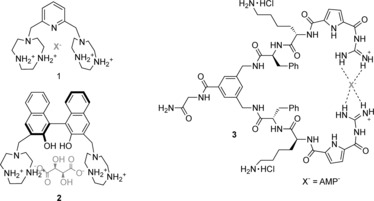
Charged ammonium and guanidinium hosts that form hydrogen bonds.

Arguably the most frequently employed motif to date is the ammonium group, used to bind anions through electrostatic and hydrogen‐bonding interactions in aqueous solution at low pH values (to ensure protonation of the amine groups),^13^ and a number of notable examples have very recently been reported. For example, Bencini and co‐workers described a series of 1,4,7‐triazacyclononane ([9]aneN_3_) macrocycles linked by rigid aromatic motifs such as compound **1** (Figure 1) that bind phosphate anions in acidic media with size selectivity arising from the distance between the polyammonium macrocyclic units.^14^ Similarly, the same research group reported a structurally related Binol‐based receptor **2** that is capable of the impressive enantiomeric recognition of the tartrate anion in aqueous buffer.^15^ The [9]aneN_3_‐derived host exhibited high selectivity in the binding and fluorescence sensing of (*S*,*S*)‐tartrate (log *K*=6.1 in 0.1 m tris‐buffer at pH 7) over (*R*,*R*)/*meso*‐tartrate, which bound approximately three orders of magnitude more weakly. Furthermore, selectivity of up to three orders of magnitude was observed over (*S*)/(*R*)‐malate, succinate, maleate, fumarate, and (*S*)/(*R*)‐lactate.

The guanidinium motif remains protonated over a much wider pH range than ammonium groups and, importantly, remains protonated at physiological pH. Furthermore, its ability to form two strong, parallel hydrogen bonds is particularly advantageous for the binding of oxoanions such as carboxylates or phosphates. For these reasons, such motifs continue to find use in receptors designed for oxoanion recognition in pH‐neutral solutions. Kuchelmeister and Schmuck recently reported a guanidinium‐based tweezers‐like receptor (**3**), which binds nucleotide anions in buffered water solution at a physiological pH value.^16^ Notably, the receptor binds monoanionic adenosine monophosphate (AMP; *K≈*10^4^ m ^−1^) with an unprecedented selectivity over trianionic adenosine triphosphate (ATP), which associates an order of magnitude more weakly.^16^ Similarly, Kataev et al. have recently reported a bisguanidinium receptor capable of the selective binding of orthophosphate in 1:1 methanol/aqueous buffer solution.^17^

**Figure 2:**
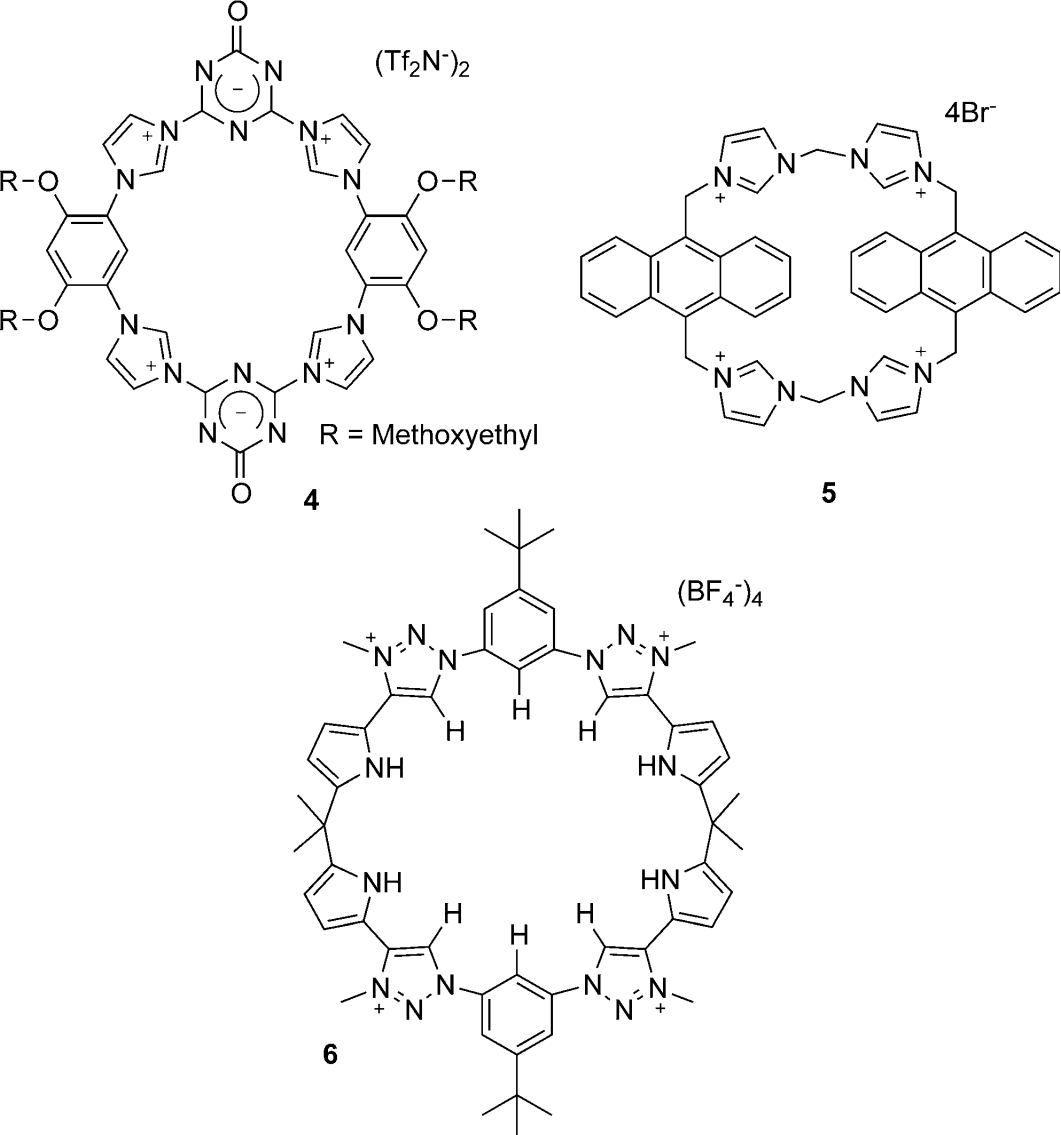
Receptors incorporating C−H hydrogen‐bond donors. Tf=trifluoromethanesulfonyl.

Although less frequently utilized for anion recognition than the ubiquitous NH hydrogen‐bond donor, C−H hydrogen bonds can be usefully employed in receptor design, and such motifs have lately been incorporated within anion receptors capable of operating in aqueous solvents. For example, You and co‐workers have described a tetrakis(imidazolium) macrocycle **4** (Figure 2) for the recognition of sulfate in water.^18^ A strong 2:1 stoichiometric complex is formed between **4** and sulfate in which the anion is bound by eight CH−O hydrogen bonds, with an association constant of 8.6×10^9^ m ^−2^. Notably, sulfate binding is concomitant with an increase in the fluorescence response of the macrocycle, thereby enabling the receptor to act as an optical sulfate sensor in aqueous solution. Kim and co‐workers have also used the tetrakis(imidazolium) cyclophane **5** for the selective detection of guanosine‐5′‐triphosphate and iodide.^19^ Binding of these species by charge‐assisted C−H hydrogen‐bonding interactions in aqueous solution at pH 7.4 results in quenching of the fluorescence from the anthracene motif. Furthermore, Hay, Sessler, and co‐workers have investigated the anion recognition properties of a pyrrole‐based triazolium‐phane **6**, which contains NH and cationic CH hydrogen‐bond donor groups.^20^ The macrocycle recognizes tetrahedral oxoanions with high affinity and selectivity over a range of monoanions and trigonal planar anions in 2:3 acetone/water solution at pH 7.2.

### 2.2 Metal Complexes

Arguably the strongest interaction that can be used for anion binding in water is direct coordination to a metal cation. Metal‐containing receptors are typically designed around an organic scaffold that binds metal cations with high kinetic and thermodynamic stability such that at least one coordination site on the metal center remains vacant or coordinated to a weakly bound substrate and thus available for anion binding. In the past five years, numerous metal‐based anion receptors have been reported and, as with examples from the more distant past, they are dominated by Zn^II^, Cu^II^, and, to a lesser extent, trivalent lanthanide cations.

**Figure 3:**
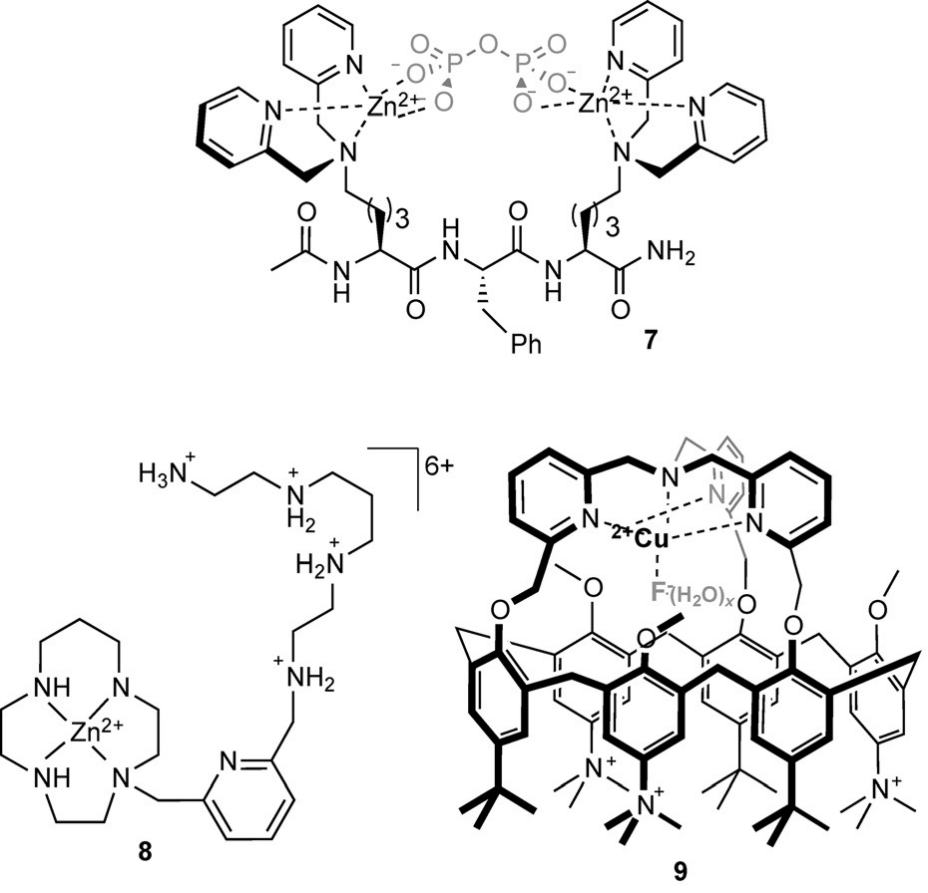
Anion receptors based on d‐metal cations.

Zn^II^‐dipicolylamine‐based receptors, such as peptide derivative **7** 21 (Figure 3), remain a popular class of compounds for the recognition of biologically relevant polyoxoanions in water. They exhibit high affinity for pyrophosphate anions in particular, and have been coupled to a range of fluorophores to facilitate applications in sensing and detection. Such receptors and their uses have been reviewed exte nsively by Jolliffe and co‐workers, and so will not be further discussed in this Review.^22^

Although direct anion to metal coordination can result in receptors exhibiting very high anion‐binding affinities, the selectivities of simple metal coordination compounds can often be poor. However, by integrating metal cations into organic scaffolds which impose structural geometric constraints and/or provide supplementary supramolecular interactions, a higher degree of guest selectivity can be realized. For example, Tripier and co‐workers have demonstrated that a dual metal‐coordination/hydrogen‐bonding approach can be effective for anion recognition in water, whereby the pendant polyammonium arm in Zn^II^ complex **8** enhances the recognition capability towards ATP and glyphosate anions.^23^

The hydrophobic effect has also be exploited to further enhance the anion‐recognition capability of metal complexes. Reinaud et al. have used Cu^II^ coordination to bind fluoride anions with high affinity (log *K*=4.9) in water at pH 5.9, by embedding the metal center within a hydrophobic cavity of a calix[6]arene **9** (Figure 3).^24^ Crucially, control experiments revealed that no binding of fluoride occurs in the absence of either the metal or the hydrophobic cavity, whilst computational studies indicate that the fluoride guest interacts with two water molecules within the cavity, which suggests that the receptor binds a partially hydrated anion. This lowers the dehydration cost of binding, whilst further stabilization may occur through OH−π interactions with the aromatic walls of the calixarene.

The use of luminescent lanthanide complexes is established as an effective approach to anion binding and sensing in water, and has recently been extensively reviewed by Butler and Parker,^25^ and so only selected recent examples are highlighted here. The binding of anions to the vacant coordination site of a Ln^III^ complex is dominated by electrostatic interactions; however, selectivity can be achieved by tuning the nature of the ligand and the lanthanide cation itself.

**Figure 4:**
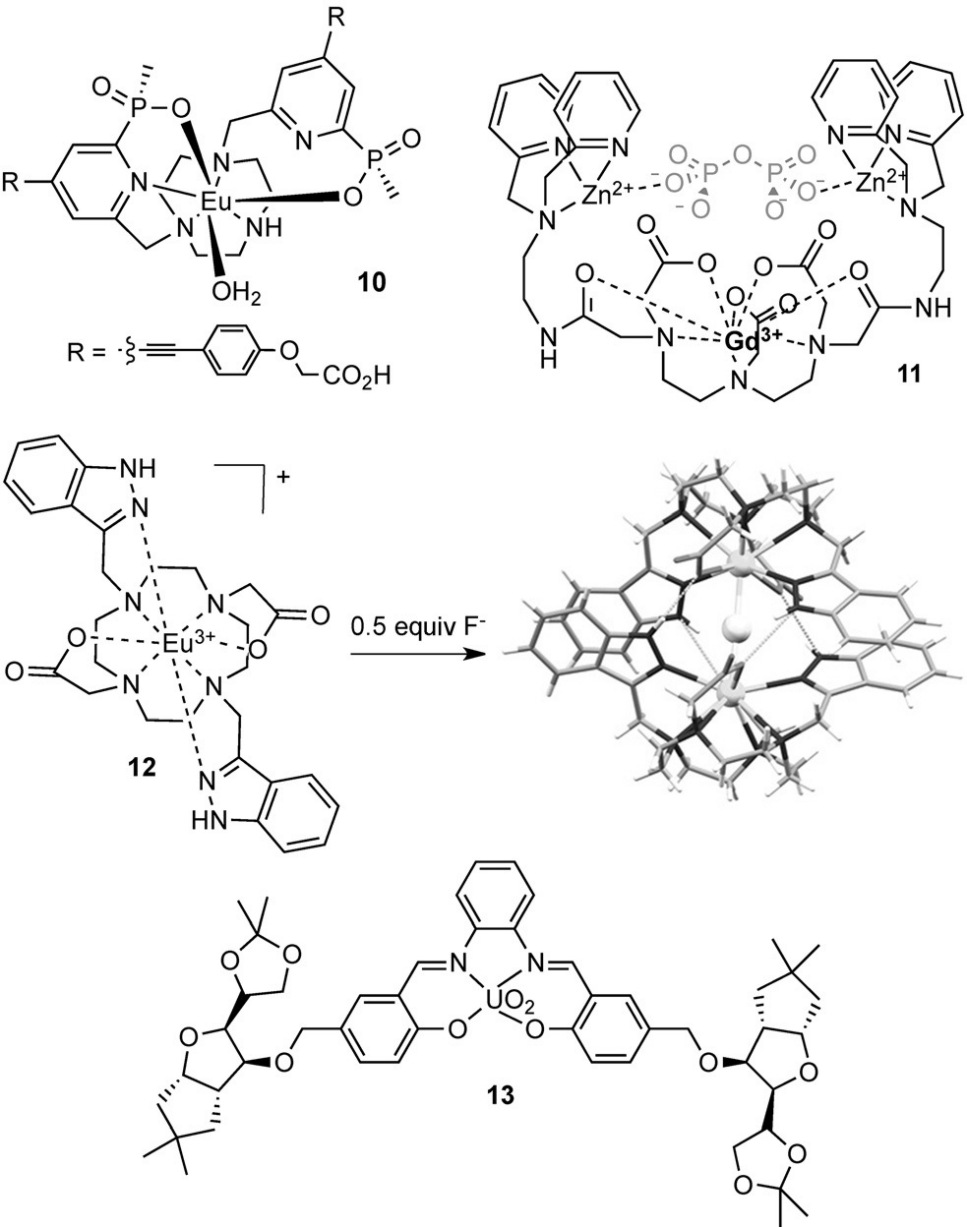
Anion receptors based on f‐metal cations.

Parker and co‐workers have recently reported a triazacyclononane–Eu^III^ complex **10** (Figure 4) which binds oxoanions at the metal center by displacement of coordinated water molecules. The complex was used to quantify bicarbonate anions in human serum by means of a ratiometric analysis of the bright europium‐based luminescence.^26^ Vilar and co‐workers have coupled a Gd^III^ complex with Zn^II^–dipicolylamine moieties to devise a pyrophosphate‐responsive magnetic resonance imaging (MRI) contrast reagent **11**.^27^ The binding of pyrophosphate anions in water solution at a physiological pH value modulates the relaxivity of the Gd^III^ complex sufficiently to be observed in imaging experiments. In contrast, a supramolecular dimerization approach has been exploited by Tripier and co‐workers to bind fluoride anions with high affinity in water.^28^ The addition of fluoride to Eu^III^ complex **12** results in the formation of a strong 2:1 host–guest complex (log *β*=13): structural data from single‐crystal X‐ray analysis reveals that, in addition to the bridging fluoride anion between the two Eu centers, the complex is further stabilized by π‐stacking interactions and hydrogen bonding. The strong fluoride association in water leads to very sensitive detection of the anion, to a limit of 0.46 ppb.

Whilst lanthanide complexes comprise the vast majority of examples of f‐metal coordination compounds for anion binding, Schiaffino and co‐workers have shown that actinide complexes can also be exploited for this purpose.^29^ They have devised a neutral, water‐soluble uranyl‐salophen complex **13** (Figure 4) that is able to bind the nucleotide polyanions ADP and ATP with high affinity (log *K*>4) in aqueous buffer solution by direct coordination to the bound UO_2_^2+^ motif.

### 2.3 Neutral and Negatively Charged Anion Receptors

The use of charged receptors is undoubtedly the primary approach used for anion recognition in water. However, the disadvantage of such charged hosts is that they rely on nondirectional electrostatic interactions which can result in poor anion selectivity, whilst accompanying counterions compete with the guest for the binding site. Neutral hosts are not affected by these disadvantages, but in general interact more weakly than their charged analogues with anions in solution, and as a result are usually confined to operating in low‐polarity organic solvents. However, in recent years several research groups have reported elegant examples of electroneutral anion receptors that are able to operate in water, by exploiting both complementary hydrogen‐bonding interactions and the hydrophobic effect to enhance the overall complex stability. Furthermore, examples of negatively charged hosts, where anion binding occurs in a central hydrophobic cavity of a receptor solubilized by peripheral appended carboxylate groups, have also been developed. Examples of neutral or negatively charged hydrophobic hosts remain uncommon; however, the area is the subject of active current interest, and in the past few years a handful of notable examples have been reported, and are discussed here.

**Figure 5:**
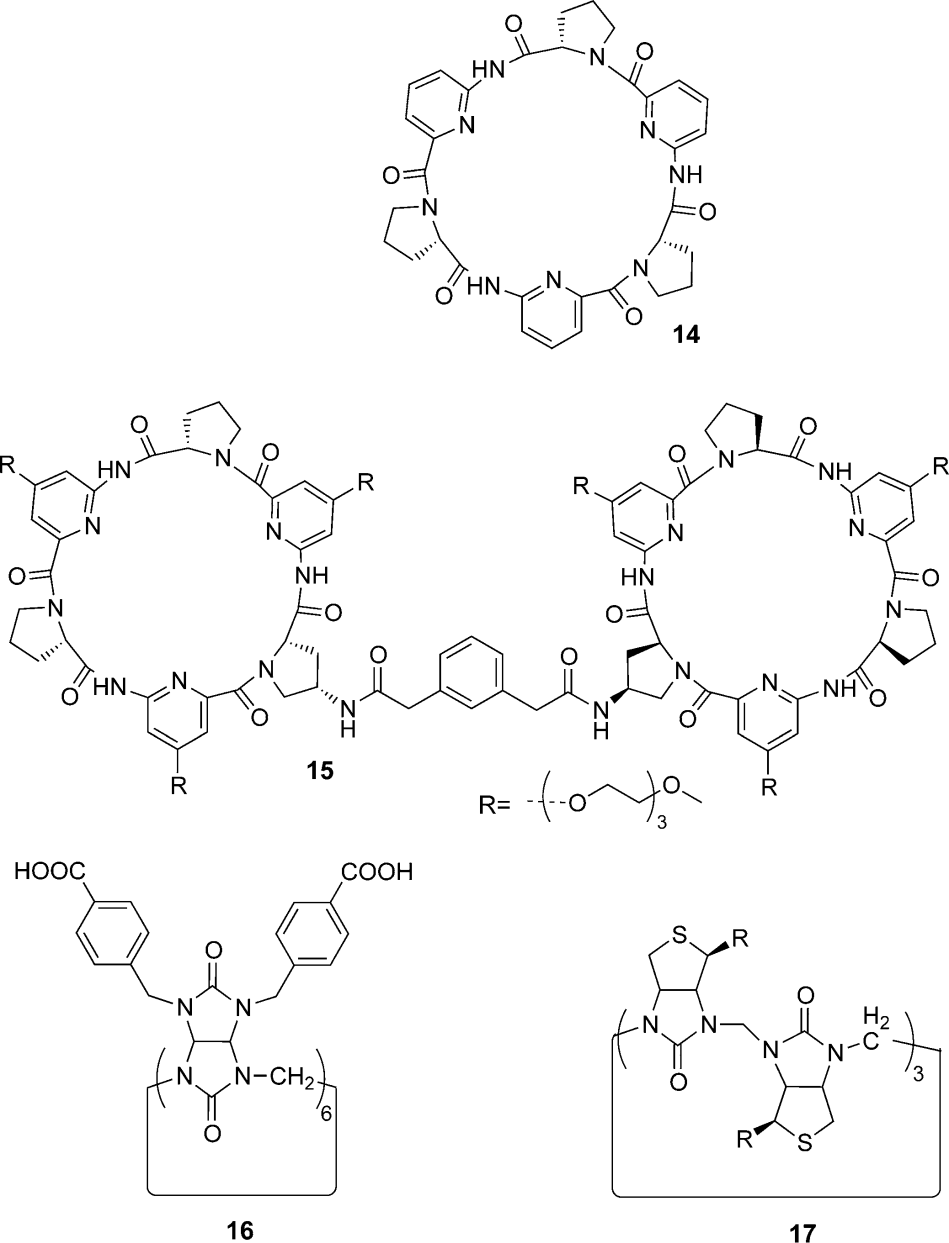
Neutral anion receptors that exploit the hydrophobic effect.

In 2001 Kubik et al. demonstrated that a cyclopeptide‐derived host **14** formed a 2:1 stoichiometric host–guest complex with halide and sulfate anions in D_2_O/CD_3_OD 80:20 solution through convergent hydrogen‐bonding interactions, aided by the hydrophobic effect (Figure 5).^30^ They subsequently showed that 1:1 host–guest stoichiometric complexes could be prepared by joining two cyclopeptide macrocycles together with a flexible linker.^31^ The same research group has now reported a related host system **15** (Figure 5) which exhibits enhanced aqueous solubility, thus enabling the recognition of iodide and sulfate in 95:5 D_2_O/CD_3_OD (log *K*=3.70 and 3.96 respectively).^32^ In both cases, a highly favorable entropic contribution is the dominant driving force for complex formation. Although not strictly belonging to this section, enhanced sulfate recognition was achieved in related cyclopeptide‐derived hosts decorated with pendent ammonium groups to enable high‐affinity binding of the anion (log *K*=5.1) in 1:1 methanol/water solution at pH 4.8, driven by both favorable entropy and enthalpy.^33^

Very recently, Sindelar and co‐workers have raised the bar on what is achievable in terms of anion‐recognition strength in water by hydrophobic host molecules, with their report of a water‐soluble variant of the bambusuril macrocycle **16**.^34^ The host binds various inorganic anions in water within the hydrophobic cavity in an enthalpy‐driven process, whilst additional stabilization of the complex arises from multiple CH−anion hydrogen‐bonding interactions. The receptors bind the anion guests with high affinity in phosphate buffer solution, with the affinity ranging from ca. 10^3^ m ^−1^ for Cl^−^, to ca. 10^5^ m ^−1^ for Br^−^ and NO_3_^−^, with the strongest binding being observed for I^−^ and ClO_4_^−^ (*K≈*10^7^ m ^−1^). The authors ascribe these remarkable affinities to the total isolation of the bound anion from water molecules by inclusion within the hydrophobic bambusuril cavity.

A related cyclic hexamer has also been shown to bind anions in water by Pittelkow and co‐workers.^35^, ^36^ They reported the halide‐anion‐templated synthesis of a macrocyclic 6+6 hexamer **17** of d‐biotin and formaldehyde, and demonstrated that the macrocycle binds a range of monovalent anions in water at pH 7.5 in phosphate buffer. The binding of softer anions such as ^−^SCN, I^−^, and SeCN^−^ is favored, with association constants ranging from log *K*=4.5 for SCN^−^ down to log *K*=1.8 for Cl^−^. Thermodynamic anion‐binding titrations using isothermal titration calorimetry (ITC) revealed that anion association in all cases is enthalpically driven, characteristic of a non‐classical hydrophobic effect in which the anion liberates high‐energy water molecules from the hydrophobic cavity of the macrocycle. Furthermore, the single‐crystal X‐ray structure of **17** with iodide provided evidence for C−H hydrogen‐bonding interactions between the host and anion.

**Figure 6:**
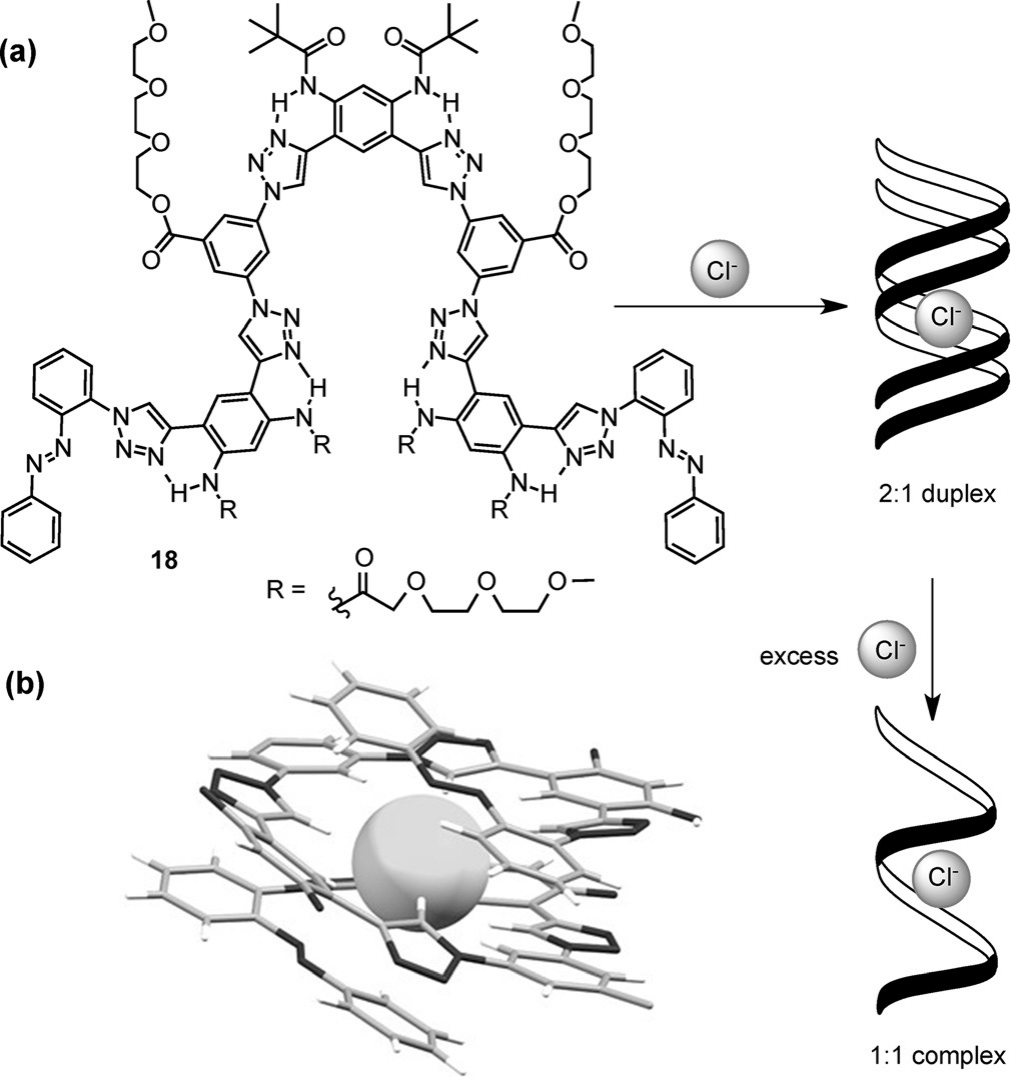
a) The chloride‐binding duplex foldamer of Flood and co‐workers; b) X‐ray crystal structure of the 1:1 stoichiometric foldamer complex with chloride (side chains removed for clarity).

The combination of C−H hydrogen bonding and the hydrophobic effect has also been elegantly used by Flood and co‐workers for the recognition of chloride anions by the aryl‐triazole foldamer **18** in organic–aqueous solution (Figure 6 a).^104^ Hydrophobic interactions are responsible for organizing and stabilizing the foldamer, which forms a 2:1 stoichiometric complex with chloride in 1:1 CH_3_CN/H_2_O solution, with about 80 % of the hydrophobic π surfaces buried within the duplex in the process. The chloride anion is bound by eleven triazole C‐H‐Cl^−^ hydrogen‐bonding interactions within a hydrophobic pocket, which shields the anion from the surrounding solvent. The formation of the chloride duplex is driven almost entirely by favorable enthalpy, with an overall stability constant of *β*_2_=9×10^12^ m ^−2^. The addition of a large excess of chloride results in the equilibrium being pushed in favor of a 1:1 stoichiometric single helix complex, the crystal structure of which is shown in Figure 6 b. In this case, only about 50 % of the hydrophobic π surfaces are buried in the foldamer, thus accounting for its lower stability than the duplex structure (*K*_1_=2×10^5^ m ^−1^). It is noteworthy that the formation of the 2:1 duplex is a highly cooperative process, with a *K*_2_/*K*_1_ ratio of 165 (where *K*_2_ is the stepwise duplex association constant, and *K*_1_ is the association constant for the 1:1 complex).

The aforementioned examples exploit the hydrophobic effect to achieve the strong binding of anions in aqueous solution. However, as previously mentioned, the hydrophobic effect is arguably the least well understood factor that drives host–guest complexation in solution. Indeed, the traditional view of describing the (classical) hydrophobic effect in terms of entropic arguments does not hold true for many examples of host–guest complexation, in which a dominant favorable enthalpy drives the association, and may be described instead by the non‐classical hydrophobic effect. The microscopic origin of the favorable negative enthalpy thermodynamic signature is ascribed to the release of high‐energy water molecules that occupy the hydrophobic binding domain of the host (and thus form fewer hydrogen bonds than in bulk water), thereby leading to the formation of more cohesive water–water interactions and a gain in enthalpy. This phenomenon was first described by Diederich and co‐workers in 1991 in the context of the recognition of arene guests by cyclophanes,^37^ but recent studies have revealed high‐affinity binding of anions in water in which the non‐classical hydrophobic effect is proposed to be the dominant thermodynamic driving force. In both the biotin hexamer reported by Pittelkow and co‐workers and the foldamer reported by Flood and co‐workers, strong anion binding is driven exclusively by favorable enthalpy: the thermodynamic signature of the non‐classical hydrophobic effect.

A related phenomenon often cited in relation to anion recognition is the Hofmeister effect.^38^ The Hofmeister effect has historically been viewed in terms of makers and breakers of water structure, originally in the context of protein solubility (kosmotropic anions increase water structure, thus enhancing the hydrophobic effect and decreasing protein solubility, whilst chaotropic anions decrease water structure, thus weakening the hydrophobic effect and increasing protein solubility). However, the role of ion–solute interactions is also important, and the nuanced relationship between these effects and anion‐recognition phenomena is only now beginning to be understood and investigated in detail.

**Figure 7:**
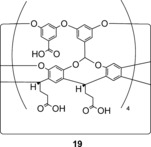
A hydrophobic cavitand anion receptor.

In an elegant recent study, Gibb and Gibb investigated the Hofmeister effect and its relationship with the hydrophobic effect in the context of anion binding within a cavitand host **19** (Figure 7).^39^ The authors demonstrated that kosmotropes, such as fluoride, acetate, and sulfate, enhanced the binding of a hydrophobic guest (adamantane carboxylate) within the cavitand, whilst chaotropes, such as perchlorate, decreased the association. This latter effect was ascribed to competitive binding of these anions within the cavitand's hydrophobic pocket. Perchlorate, in particular, was shown to bind surprisingly strongly (*K*_a_=95 m ^−1^), and a more recent investigation has shown that the binding of the anion is dependent on both the nature and concentration of co‐salts.^40^ The overall trend (in which chaotropic anions decreased perchlorate anion association whilst kosmotropic anions enhanced it) directly reflects the Hofmeister series. The authors proposed that the former may be explained in terms of competitive binding of relatively poorly hydrated and polarizable anions leading to an overall decrease in perchlorate association, whilst, in contrast, strongly hydrated salts enhance anion association as a result of cation binding to the cavitand's peripheral carboxylate groups and thus reduce the net negative charge of the host. These results demonstrate an unprecedented molecular insight into the role of ion–solute interactions in the Hofmeister effect, and provide a link between this hitherto ambiguous concept and those of hydrophobicity and anion binding.

The binding of chaotropic anions within hydrophobic cavities has also recently been explored by Nau and co‐workers, who investigated the binding of negatively charged dodecaborate clusters of the form B_12_X_12_^2−^ and B_12_X_11_Y^2−^ (where X=H, Cl, Br, and I; and Y=OH, SH, NH_3_^+^, and NR_3_^+^) within γ‐cyclodextrin.^41^ Such clusters were found to bind with remarkably high affinity (log *K*=3–6), but with low selectivity between anions of differing size, and driven exclusively by a strong enthalpic contribution to the binding whilst disfavored by entropy. The authors ascribe these data to two effects: 1) increased dispersion interactions between the anion and the cyclodextrin compared to those between the anion its solvation shell, and 2) the so called “chaotropic effect”, in which the borate clusters act as chaotropic anions and disrupt the local water structure. Following the latter argument, the negative complexation enthalpy is explained by the re‐formation of the broken hydrogen bonds in the bulk aqueous solution upon binding of the anion in the hydrophobic cavity. The negative entropy change arises from the loss of structural entropy in the bulk water upon binding of the cluster and the subsequent re‐formation of hydrogen bonds. This recovery of hydrogen bonds as a supramolecular driving force is analogous to the common description of the non‐classical hydrophobic effect, such as in the case of anion binding by the aforementioned biotin cyclic hexamer **17**, where the release of high‐energy water molecules from within the macrocyclic cavity, as opposed to those surrounding the chaotropic anion, is driven by the enthalpically favorable re‐formation of hydrogen bonds with the bulk solvent. This study highlights the need for considering in detail the solvation of both the guest and host, and the influence of high‐energy water molecules within both the host cavity and surrounding a chaotropic anion on the thermodynamics of hydrophobic‐driven anion binding in water.

### 2.4 Halogen‐Bonding Anion Receptors

**Figure 8:**
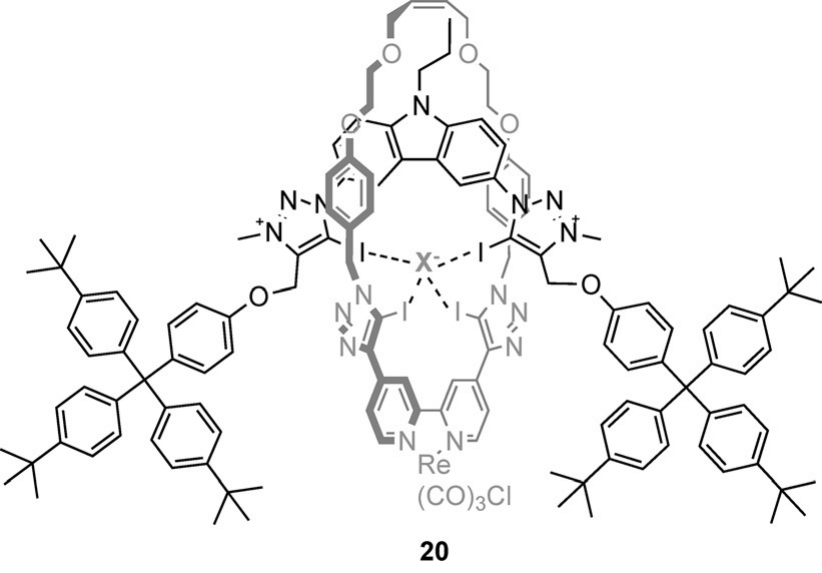
Re^I^‐functionalized halogen‐bonding rotaxane.

Halogen bonding,^42^ the attractive interaction between an electron‐deficient halogen atom and a Lewis base, has in recent years attracted much interest as an intermolecular interaction analogous to the ubiquitous hydrogen bond.^43^ Whilst extensively utilized in the solid state, its application in the solution phase is less developed, but has recently been shown to be an effective interaction for anion recognition in organic solvents.^44^ However, it was only in the last year that seminal examples of halogen‐bonding receptors capable of anion recognition in water were developed. In 2014, we reported a rotaxane host **20** capable of exclusively binding halide anions over a range of oxoanions in aqueous organic solvent mixtures through four convergent halogen bonds in the cavity formed between the interlocked components (Figure 8).^45^ An incorporated luminescent rhenium(I) bipyridyl motif in the macrocycle facilitated the study of the anion‐binding properties of the rotaxane by allowing the change in the Re^I^ metal‐ligand‐charge‐transfer (MLCT) emission to be monitored. This revealed a selectivity trend of I^−^>Br^−^>Cl^−^ in 1:1 CH_3_CN/H_2_O solution, with remarkably high affinity for a doubly charged receptor (log *K*_a_=4.4, 3.8, and 2.7, respectively).

**Figure 9:**
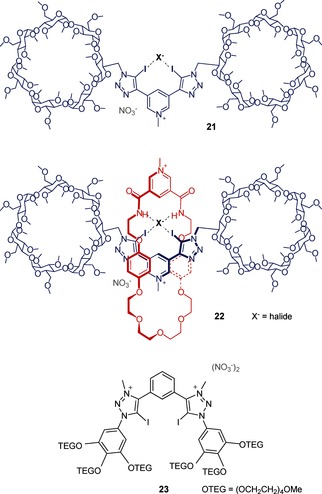
Water‐soluble halogen‐bonding anion receptors.

We later published a seminal study to quantify halogen‐bonding interactions in 100 % water, and demonstrated that the intermolecular interaction is superior to hydrogen bonding for anion recognition in aqueous solution.^46^ Anion‐binding titrations in D_2_O with a series of acyclic and rotaxane‐based receptors containing the bidentate 3,5‐bisiodotriazole pyridinium halogen bond donor motif, and solubilized with permethylated β‐cyclodextrin derivatives, revealed a remarkable enhancement in the binding affinity for halide anions when compared to analogous C−H and amide N−H hydrogen‐bond donors. For example, XB rotaxane **22** (Figure 9) binds iodide with high affinity in water (*K*_a_=2200 m ^−1^), two orders of magnitude more strongly than the analogous 3,5‐bisprototriazole pyridinium C−H hydrogen‐bonding rotaxane system (*K*_a_=40 m ^−1^). Remarkably, the halogen‐bonding acyclic receptor **21** is also capable of anion recognition in water despite its simplicity and low charge. Van‘t Hoff analysis of the iodide recognition process by XB‐rotaxane **22** revealed that the binding is enthalpically driven. This is in contrast to the analogous rotaxane hydrogen‐bonding systems, in which the association is driven by entropy, thus reflecting both the charge‐transfer contribution to XB–anion interactions^47^ and differing desolvation thermodynamics of XB donors that contribute to the remarkable anion‐binding properties. A later study, in which the solubilizing β‐cyclodextrin derivatives of a hydrogen‐bonding rotaxane were modified, revealed that these stoppers play a minimal contribution to the overall anion‐binding affinity of the host in water, thus suggesting that a wider range of solubilizing stopper groups may be employed without adversely affecting the anion‐recognition capability of the host system.^48^ Indeed, we have since employed polyethylene glycol derivatives to solubilize a bisiodotriazolium‐based acyclic halogen‐bonding receptor **23**, which is capable of enhanced perrhenate recognition in D_2_O compared to the hydrogen‐bonding analogue, which is again enthalpically driven.^49^

## 3 Macromolecular Recognition of Anions in Water

Small‐molecule receptors have dominated anion‐binding and anion‐sensing technologies to date, largely because of the potential for the direct rational design and simple delineation of structure–function relationships. However, there is much to be said for macromolecular and nanotechnological approaches to anion recognition, particularly when faced with the challenges of aqueous media. Larger systems allow the construction of solvent‐excluded microdomains to maximize binding strength, the exploitation of multivalency and cooperative recognition, signal amplification, and fine control of material properties such as solubility and rheology.

With the exception of a handful of small‐molecule anion transporters such as prodigiosin, it is macromolecules which have been selected in nature to perform highly selective anion‐binding tasks. In recent years there have been a number of important advances in the study of anion binding by biomacromolecules, which we will summarize in Section 3.1; the discussion will not be exhaustive (the number of proteins which bind ATP makes that task somewhat herculean), but we will consider the most significant advances from the viewpoint of supramolecular design.

### 3.1 Biomolecules

Living systems must manipulate anions in water in a huge number of sophisticated ways and, therefore, biology is an important point of reference for anion recognition in water.

**Figure 10:**
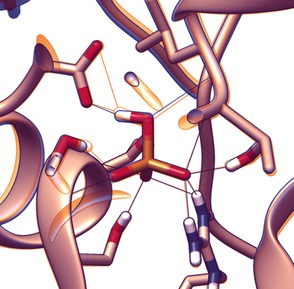
Binding environment of hydrogen phosphate (orange) and hydrogen arsenate (blue) by GFAJ‐1 phosphate‐binding protein (PDB 4F19 and 4F18, respectively). Note the clamping of unprotonated oxo groups, which causes the protonated oxygen atom to be maximally displaced, and subsequent distortion of hydrogen‐bonding geometry at the protic site of the anion.

The scientific response to the apparent finding of bacteria (GFAJ‐1) living in an arsenic‐rich, phosphate‐free environment,^6^ and suggesting the existence of arseno‐DNA, was comprehensive: chemists noted that the rates of hydrolysis^50^ and reduction^51^ of arsenate esters would predict a prohibitively low stability for arseno‐DNA; furthermore, biologists showed that trace levels of phosphate were present and sufficient for bacteria,^7^ and found no detectable arsenic in their DNA.^52^ It transpired that the bacteria are able to tolerate high arsenate/low phosphate environments because they can be extremely selective in their uptake of the two: a phosphate‐binding protein (PBP) up‐regulated by GFAJ‐1 in phosphate‐poor conditions has a 4500‐fold preference for phosphate over arsenate. This is especially striking considering that the sizes of the anions differ by only 4 %, their p*K*_a_ values are nearly identical, and their oxygen atoms are very similarly charged. The molecular basis for this discrimination was elucidated the following year^53^ by Elias, Wellner et al., who discovered that a network of dipole–anion interactions and repulsive interactions were responsible. Both phosphate and arsenate anions are bound in their monoprotonated forms, and the locations of the three nonprotonated oxygen atoms are firmly anchored, thus maximizing the directionality of the size difference (Figure 10). In the case of arsenate, this results in a steric clash between the protonated oxygen atom with two Cβ atoms on nearby residues and distorts a low‐barrier hydrogen bond between the OH group of the anion and a proximal aspartate side chain. Conversely, all these parameters are optimal when the cleft is occupied by phosphate. This astounding example of natural selectivity serves as a gold standard for synthetic anion hosts: it is possible not just to bind anions strongly in water, but to have total control over selectivity between very similar targets.

**Figure 11:**
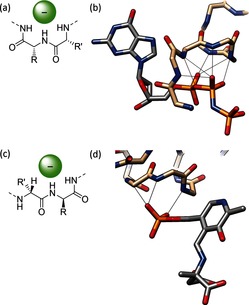
a) Simplified illustration of the most basic nest motif. b) GTP analogue GppNp bound by hydrogen bonds from five consecutive main‐chain amides in a nest formation (PDB 5P21^56^). c) Simplified illustration of the C^α^NN motif. d) Binding of a phosphate group by a C^α^NN motif located at the end of a helix (PDB 1AJS^57^).

Another important finding for anion‐binding proteins was the identification by Watson and Milner‐White of a particular fold, named the “nest”, which can occur with a minimum of just two residues (Figure 11 a).^54^ The nest is described by residues with alternating main‐chain dihedral angles of *φ*,*ψ*≈−90,0° and *φ*,*ψ*≈70,40° (or their negative). When two adjacent residues display this conformation, an atom‐sized concave space is formed into which the two main‐chain NH groups plus that of the following amino acid point, thus creating a space suitable for coordination of an anionic or δ^−^ atom (Figure 11 a). The arrangement and spacing of NH groups is analogous to that of oxygen atoms in crown ethers. This observation generalized a number of previously known specific anion‐binding motifs such as the alpha turn and the paperclip/Schellman loop.^55^

Compound nests consist of longer tracts of residues displaying these dihedral angles and results in larger cavities such as those known as P‐loop, which is the most commonly found ATP or guanosine triphosphate (GTP) binding motif in proteins and contains a four‐residue nest (Figure 11 b). In serine protease, another four‐residue nest is used to stabilize a tetrahedral oxyanion intermediate, while nests of up to eight residues are often found supporting iron–sulfur clusters.^58^ These observations have led to new anion hosts, a first example of which is a helical hairpin eicosapeptide which was found to crystallize (albeit from organic solvents) with an included acetate anion in a nest bridging the two helices,^59^ and in 2012 a minimal hexapeptide was designed that contained a P‐loop‐type nest which was shown to bind HPO_4_^2−^ strongly in water at neutral pH.^60^ Interestingly, it appeared that a deprotonated amine from a lysine side chain accepts a hydrogen bond from the phosphate, as seen in phosphate‐binding proteins.

A similar anion‐recognition moiety to the nest is the C^α^NN motif, which consists of two main‐chain amide hydrogen bonds and a C−H hydrogen bond from the next α‐carbon atom (Figure 11 c).^61^ The system was initially put forward as a phosphate‐recognition unit (Figure 11 d), but by attaching Leu‐Gly‐Lys‐Gln, a C^α^NN fragment, to the N terminus of an anchor helix, Sheet and Banerjee observed sulfate recognition in water manifested as a change in the conformation that was typical of an “induced fit” system.^62^ Computational analysis confirmed earlier crystallographic studies showing that both sulfate and phosphate binding triggers a conformational switch from a nonhelical to a helical morphology.^63^

In addition to these findings, there have been a number of incremental discoveries related to the binding of oxoanions^64^ which reveal interesting aspects of natural anion‐coordination strategies. The binding of nitrate by cyanobacteria provides nitrogen fixation for entire aqueous ecosytems. In the open ocean, such ions are scarce, and so the cyanobacteria need highly evolved capture systems. In NrtA, a nitrate binding protein which is tethered to the extracellular side of the cyanobacterial membrane as part of a transporter system, nitrate is bound in a distinctly asymmetric fashion.^65^ While all three of the nitrate oxygen atoms are indistinguishable in water, when bound, the charge is localized on just one oxygen atom, with complementary positively charged groups focused about just that atom. The other two oxygen atoms are more weakly hydrogen bonded, with one in a markedly hydrophobic cleft, thereby enabling the protein to also host the nitrite anion. The system is highly homologous with a cyanobacterial bicarbonate binding protein, CmpA, which may also relate to its asymmetric coordination mode.^66^ Another nitrate receptor was discovered as part of the nitrate regulatory element (Nre) of staphylococci, which enables the bacteria to reduce the oxoanion instead of oxygen during anaerobic respiration. The binding pocket is primarily hydrophobic, provides only four hydrogen bonds, and could also host iodide anions; unusually, the protein's biological function could also be activated in the presence of iodide.^67^ It was discovered in 2009 that bacterial uptake of molybdate and tungstate was enabled through modification of the anion itself: up to that time all known metalloprotein structures containing Mo or W displayed tetracoordinate metal centers, but octahedral coordination was encountered in transporter proteins, with two carboxylate groups providing extra coordination directly to the metal centers while hydrogen‐bond donors complemented the oxygen atoms.^68^ Such a finding could provide inspiration for new strategies to bind multiatomic anions. Motivated by the possibility of noncovalent encapsulation of radionuclides for nuclear medicine, the molybdate‐binding protein has been re‐engineered to show selectivity for perrhenate as a model for pertechnetate (for ^99m^Tc) and for β‐emitting ^188^Re and ^186^Re in its own right.^69^

Nucleic acids, the other major class of biopolymer, may seem unlikely candidates for anion coordination because of the multiply negatively charged phosphate backbone, but there are some interesting possibilities raised by the prospect of aptamers. Nucleic acid aptamers are single‐stranded oligonucleotides which fold in a particular way to bind a specific substrate^70^ in aqueous media (nucleic acids being poorly soluble in anything else). Charge screening required for folding and recognition is achieved through the addition of inorganic cations. Aptamers are alternatives to antibodies, and can be “raised” through a more simple in vitro selection process against any potential target,^71^, ^72^ ranging from monoatomic ions through small molecules and proteins, up to whole cells. To date they have been only sparsely explored in anion recognition, despite examples of metal‐ion binding being quite numerous.^73^, ^74^ Aptamers for nucleoside phosphates are well known.^75^ In particular, an ATP aptamer has been discovered which is highly selective for the longer oligophosphates over the shorter analogues, which shows that nucleic acids can even bind polyanions strongly and selectively (in the presence of Mg^2+^ ions).^76^ An aptamer for AMP has been coupled to fluorescent nanoparticles, thereby providing a highly selective sensor for that target.^77^ Besides this, aptamers have been reported for biologically relevant anionic porphyrins,^78^ again highlighting the possibilities for further exploitation in aqueous anion‐recognition chemistry. Very recently, DNA‐like oligomers with synthetic units replacing nucleobases were reported for pattern‐based detection of anion pollutants in water.^79^ A library of 1296 tetrameric strands was created on beads from six monomers, including two metal‐binding ligands and three organic fluorophores. Metals were added to provide binding sites, and the fluorescent response of the beads examined using 17 anions (including underexamined examples such as selenite, arsenate, bromate, and permanganate) in aqueous buffer solution. The most responsive systems were selected and narrowed down to a set of eight, which in combination could discriminate between all the anions and measure their concentrations at micromolar concentrations.

### 3.2 Liposomes and Membrane Transport

**Figure 12:**
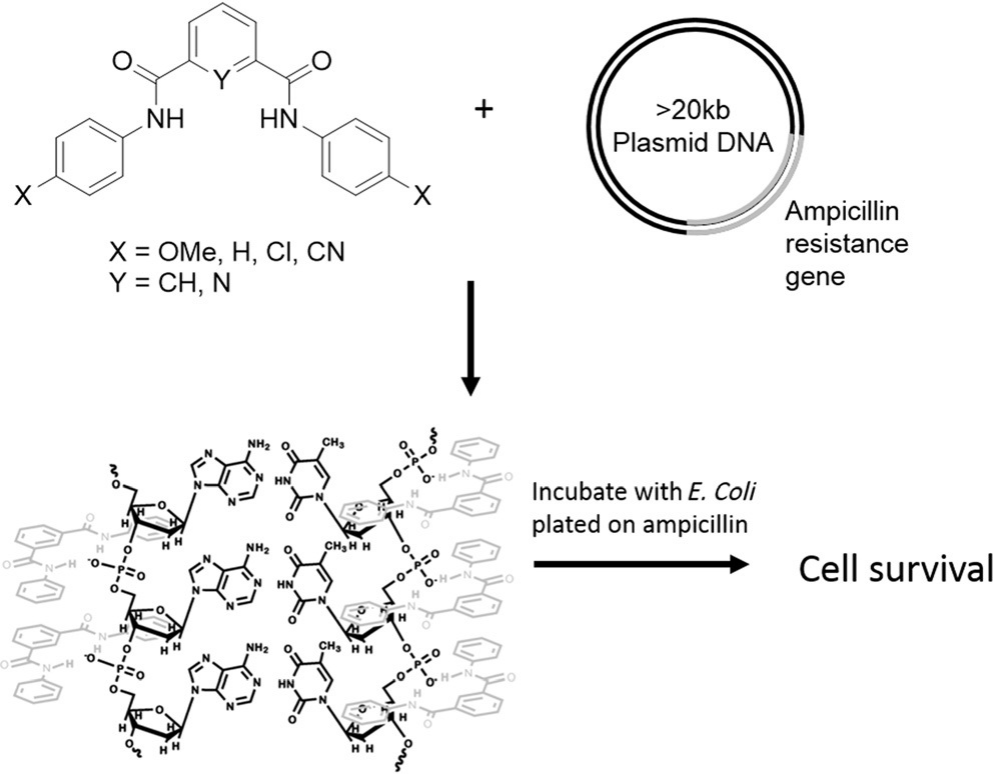
Transfection of DNA macromolecules across cell membranes for genetic modification using small anion hosts. Adapted from Ref. 87 with permission. Copyright 2012 American Chemical Society.

Biological anion binding frequently relates to cross‐membrane transport, and synthetic mimicry of anion binding is, therefore, often motivated by related biological and medicinal possibilities. Transmembrane anion channels^80^ have been made, as well as transporter systems, largely led by Gale and co‐workers.^81^, ^82^, ^83^ In these studies, an anion host (typically an uncharged small molecule) is added to a buffered aqueous solution containing liposomes with an anion concentration gradient imposed across the membrane. The resultant transport across the membrane is then measured. It is currently an open question as to whether anion coordination occurs in water with the anionophores freely entering the aqueous phase (where some can certainly be present^83^) or at the membrane interface with transporters confined within the leaflets. Some studies report the strength of the anion–host interaction, but only in low‐water media.^84^ The mobility of the anionophore likely relates to lipophilic balance^83^—the transporter must be able to get through the hydrophobic membrane, while still bind the anion—and also potential for self‐assembly within the bilayer to give channels.^85^, ^86^ Whatever the case, the entire supramolecular system can be viewed as an anion host. One outstanding example of this area is use of small molecular isophthalamides for DNA transfection by Gokel and co‐workers (Figure 12).^87^ By employing a very simple anion host system, it was possible to transfect plasmids of >20 kbase with ampicillin resistance across the membrane into bacterial cells, thereby resulting in robust colony formation. Importantly, the plasmids transfected were larger than those previously thought possible by chemical means.

### 3.3 Polymers

**Figure 13:**
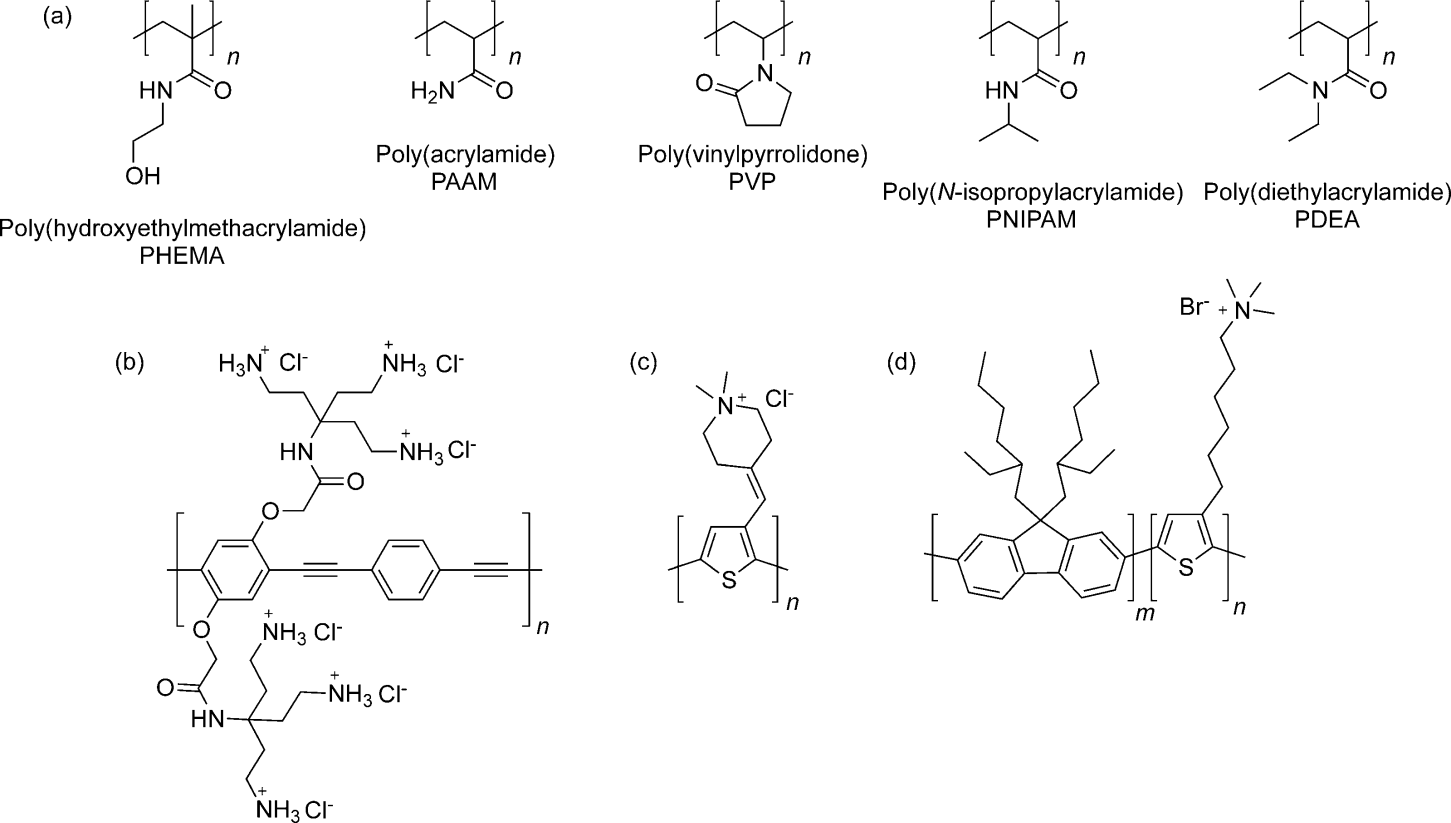
Polymers used in aqueous anion recognition. a) Polyolefins. b) Ammonium‐appended poly(phenylene‐ethynylene). c) Ammonium‐appended polythiophene. d) Ammonium‐appended poly(fluorene‐*b*‐thiophene).

Polymers are particularly attractive for recognition applications as a consequence of their multivalent effects, creation of microdomains, tunability of solubility and physical properties, and processability for materials production. Despite these advantages, the field is largely undeveloped. Foundational studies on the interaction of anions with polyolefins were carried out a quarter of a century ago, when Jhon and co‐workers established that NMR line broadening of the ^127^I signal in water in the presence of polymers such as PHEMA, PAAM, and PVP was due to binding (Figure 13 a).^88^ In the case of PVP, the binding strength was found to correlate with the Hofmeister series, with the more chaotropic anions interacting most strongly.^89^ These studies were picked up only in the last ten years with PNIPAM, which is a lower critical solution temperature (LCST) polymer, meaning that it is insoluble in water above a certain temperature. Cremer and co‐workers found that that the transition temperature was lowered by more hydrated (i.e. kosmotropic) anions and correlated with the hydration entropy.^90^ Less hydrated anions (ClO_4_^−^>SCN^−^>Br^−^>NO_3_^−^), on the other hand, actually salted in the polymer at low concentration, thereby raising the transition temperature, while at high concentrations the increased surface tension resulted in salting out. Analysis of the concentration/LCST curves revealed a component of direct anion binding was responsible for the improved solubility, which was presumed to be occurring through the amide group as a hydrogen‐bond donor. The structural basis for salting‐in was analyzed in more detail using PDEA, an LCST polymer with a tertiary rather than secondary amide, and hence no apparent anion‐binding site.^91^ Combined NMR and IR spectroscopic studies showed that the same chaotropic anions were interacting with the α‐CH proton on the polymer backbone. While this may seem unlikely at first glance, it is consistent with the involvement of α‐CH peptide protons in anion binding, as in the C^α^NN motif (see Section 3.1).

The utilization of polymers for anion recognition in water has centered on exploitation of the photophysical properties of conjugated systems for sensing,^92^ for the most part by inducing the kind of desolvation discussed above. For example, by decorating poly(phenylene‐ethynylene) with cationic primary ammonium side chains (Figure 13 b), it is possible to sense pyrophosphate through a recognition‐induced aggregation mechanism that gives a ratiometric fluorescent response.^93^ Conversely, poly(thiophene) decorated with pendant quaternary ammonium groups (Figure 13 c) disaggregates in water in the presence of iodide and sulfonate‐based surfactants to give a red‐to‐yellow color change with a sensitivity down to the nanomolar regime.^94^ Poly(fluorene‐*b*‐thiophene) has also been functionalized with tertiary ammonium groups on the thiophene block (Figure 13 d), and the fluorescence of that section was found to quench with halides in water (Cl^−^>Br^−^>I^−^), although no change of aggregation state was observed in this case.^95^ By using the constant fluorescence of the poly(fluorene) block, it was possible to measure DNA concentrations down to the sub‐micromolar level.

### 3.4 Nanomaterials

**Figure 14:**
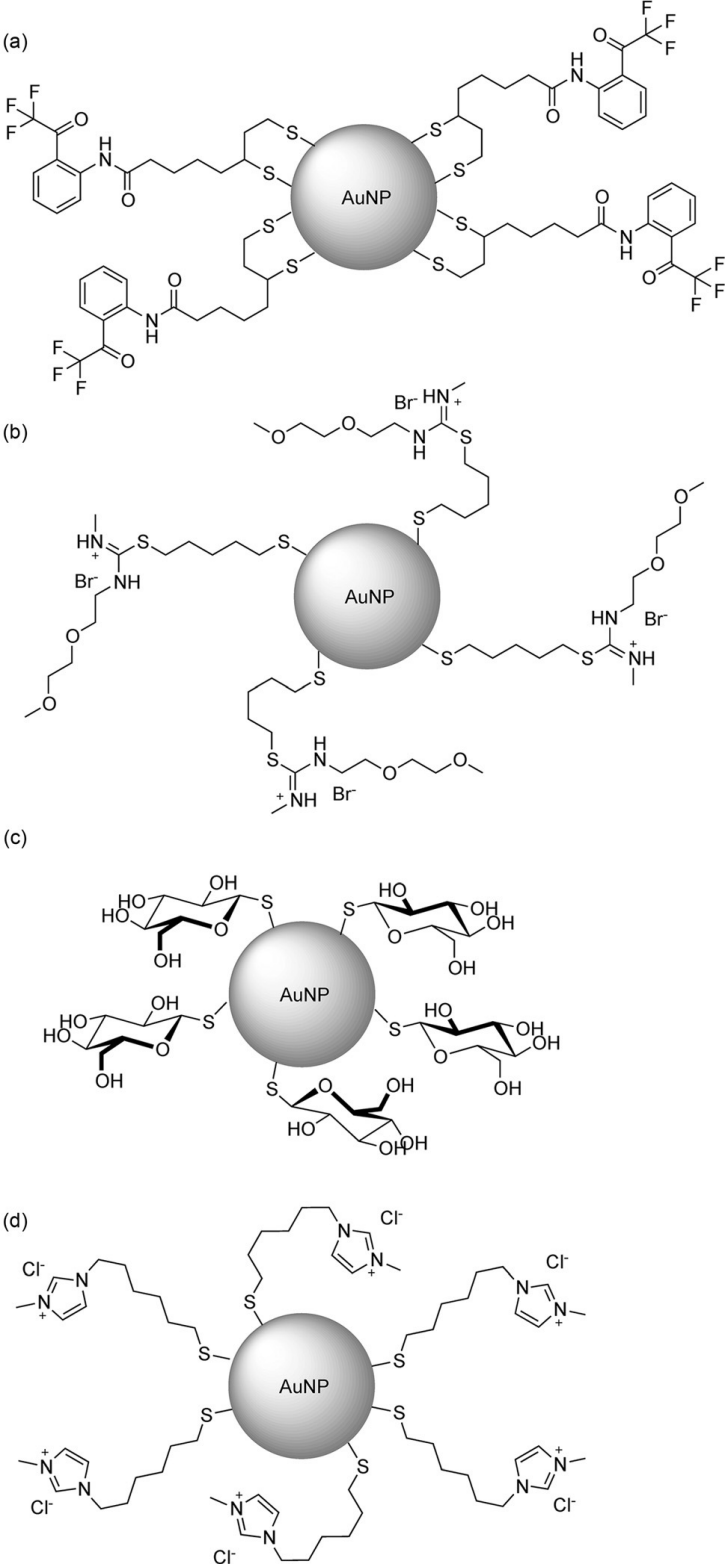
Ligands used to activate gold nanoparticles for anion recognition in water.

Aggregation‐induced spectral changes also dominate examples of synthetic nanomaterial systems for anion recognition in water. The plasmon resonance band of gold nanoparticles (AuNPs) is highly sensitive to the aggregation state, and by using anions to bring nanoparticles together, it is possible to construct sensors. Amide ligands grafted onto poly(vinyl alcohol)‐stabilized AuNPs (Figure 14 a) result in discrimination between the isomeric dicarboxylates fumarate and maleate in water: the former give a purple solution caused by bunching of the particles, whereas other dicarboxylates give red solutions.^96^ Aqueous solutions of thiouronium‐decorated AuNPs (Figure 14 b) also give selective aggregation‐induced color changes in the presence of hydrophobic anions. In this case, an interesting approach was used to generate a fluoride sensor by adding a phenylboronic acid to the solution and sensing the resultant fluoroborate.^97^ Apparently, direct fluoride‐induced aggregation was seen with thioglucose‐capped AuNPs in buffer (Figure 14 c).^98^ Cationic imidazolium ligands on AuNPs (Figure 14 d) gave strong aggregation in the presence of hydrophobic anions such as tetrafluoroborate and hexafluoroacetate, thereby making the constructs insoluble in water and facilitating phase transfer into ionic liquid media.^99^ The use of the same ligands on magnetic iron oxide NPs enabled DNA catch‐and‐release through stepwise anion exchange: starting chloride anions were displaced by DNA, which could then be released through displacement by hydrophobic anions.^100^ Similarly, imdazolium‐functionalized multiwalled carbon nanotubes are soluble in water only in the presence of chloride, thus providing an anion‐controlled aggregation switch.^101^

Although there are few examples of synthetic macromolecules for anion recognition in water, there are further types of nanosystems which have not yet been explored at all. Dendritic systems, for example, have yet to be explored. This is despite the systems potentially displaying the “dendritic effect” that gives rise to signal amplification, as observed by Astruc and co‐workers, in less‐water‐soluble ferrocene‐based systems.^102^, ^103^ The whole range of nanocarbons has barely been touched, while nanoparticles of non‐gold elements are also virgin territory. There is vast scope in these fields for researchers aiming to create potent and functional materials for the challenge of anion recognition in water.

## 4 Summary and Outlook

The findings surveyed above clearly illustrate that a number of approaches can now be exploited to achieve the recognition of anions in water. Effective strategies both for the binding of guests and solubilization of small‐molecule hosts have been outlined. Some are obvious, such as charge, but others such as the hydrophobic effect require much more in‐depth research to fully understand. New anion‐binding methods such as utilizing CH hydrogen bonds and halogen bonding feed into this theme and allow the creation of hydrophobic, solvent‐separated binding domains for fine‐tuning the recognition through control of the microenvironment. The world of biomolecules continues, as ever, to provide new insights and inspiration for tuning selectivity, while polymers and nanomaterials are just starting to emerge as promising candidates for anion extraction and sensing materials of tomorrow. Most importantly, as aqueous anion coordination chemistry becomes more mainstream, many of the long‐term proposed applications in the industrial, environmental, biological, and medical arenas will develop into real‐life technologies.

## Biographical Information

*Matthew Langton graduated from Lincoln College*, *University of Oxford, in Chemistry (MChem) in 2011 and completed his DPhil in 2014*, *under the supervision of Prof. Paul Beer. He remained in the Beer research group as an EPSRC Doctoral Award Postdoctoral Researcher*, *before moving to the University of Cambridge in 2015 to take up an Oppenheimer Early Career Research Fellowship*, *based in the group of Prof. Chris Hunter FRS. His research interests focus on supramolecular chemistry*, *with particular emphasis on the template‐directed synthesis of interlocked molecules*, *anion recognition and sensing*, *and molecular recognition through halogen bonding*.

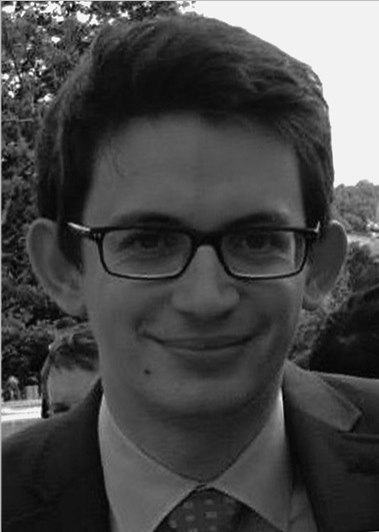

## Biographical Information

*Christopher Serpell completed his undergraduate studies in 2005 at the University of Oxford. He undertook his DPhil studies in Prof. Paul Beer's group with EPSRC funding. In 2011 he moved to McGill University as a Tomlinson and then a Banting Fellow in Prof. Hanadi Sleiman's group. He then took up a Marie Curie Fellowship in 2014 with Prof. Benjamin Davis FRS at the University of Oxford and was appointed Lecturer in Chemistry at the University of Kent in July 2015. His research interests are centered on the confluence of supramolecular and nanoscale chemistry*.

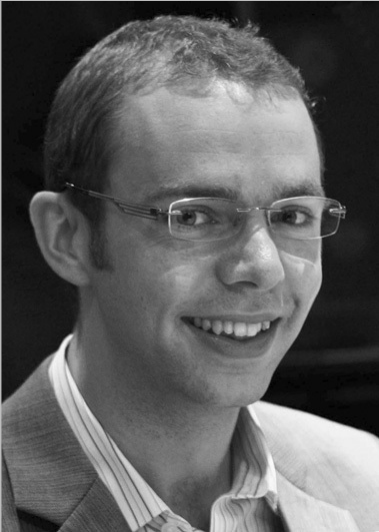

## Biographical Information

*Paul Beer obtained a PhD from King's College London in 1982 with Dr. C. Dennis Hall. After a Royal Society European Postdoctoral Fellowship with Professor J.‐M. Lehn and a Demonstratorship at the University of Exeter*, *he was awarded a Lectureship at the University of Birmingham in 1984. In 1990*, *he moved to the University of Oxford*, *where he was made a University Lecturer and Tutorial Fellow at Wadham College*, *and became a Professor of Chemistry in 1998. His research interests include coordination and supramolecular chemistry*.